# Functional Genetic Variants in *DC-SIGNR* Are Associated with Mother-to-Child Transmission of HIV-1

**DOI:** 10.1371/journal.pone.0007211

**Published:** 2009-10-07

**Authors:** Geneviève Boily-Larouche, Anne-Laure Iscache, Lynn S. Zijenah, Jean H. Humphrey, Andrew J. Mouland, Brian J. Ward, Michel Roger

**Affiliations:** 1 Laboratoire d'Immunogénétique, Centre de Recherche du Centre Hospitalier de l'Université de Montréal (CRCHUM), Montréal, Canada; 2 Département de Microbiologie et Immunologie, Université de Montréal, Montréal, Canada; 3 Department of Immunology, College of Health Sciences, University of Zimbabwe, Harare, Zimbabwe; 4 Department of International Health, Johns Hopkins Bloomberg School of Public Health, Baltimore, Maryland, United States of America; 5 Department of Medicine, The Lady Davis Institute for Medical Research and McGill AIDS Center, McGill University, Montreal, Canada; 6 Research Institute of the McGill University Hospital Complex, Montreal, Canada; BMSI-A*STAR, Singapore

## Abstract

**Background:**

Mother-to-child transmission (MTCT) is the main cause of HIV-1 infection in children worldwide. Given that the C-type lectin receptor, dendritic cell-specific ICAM-grabbing non-integrin-related (DC-SIGNR, also known as CD209L or liver/lymph node–specific ICAM-grabbing non-integrin (L-SIGN)), can interact with pathogens including HIV-1 and is expressed at the maternal-fetal interface, we hypothesized that it could influence MTCT of HIV-1.

**Methods and Findings:**

To investigate the potential role of DC-SIGNR in MTCT of HIV-1, we carried out a genetic association study of DC-SIGNR in a well-characterized cohort of 197 HIV-infected mothers and their infants recruited in Harare, Zimbabwe. Infants harbouring two copies of DC-SIGNR H1 and/or H3 haplotypes (H1-H1, H1-H3, H3-H3) had a 3.6-fold increased risk of in utero (IU) (P = 0.013) HIV-1 infection and a 5.7-fold increased risk of intrapartum (IP) (P = 0.025) HIV-1 infection after adjusting for a number of maternal factors. The implicated H1 and H3 haplotypes share two single nucleotide polymorphisms (SNPs) in promoter region (p-198A) and intron 2 (int2-180A) that were associated with increased risk of both IU (P = 0.045 and P = 0.003, respectively) and IP (P = 0.025, for int2-180A) HIV-1 infection. The promoter variant reduced transcriptional activity *in vitro*. In homozygous H1 infants bearing both the p-198A and int2-180A mutations, we observed a 4-fold decrease in the level of placental DC-SIGNR transcripts, disproportionately affecting the expression of membrane-bound isoforms compared to infant noncarriers (P = 0.011).

**Conclusion:**

These results suggest that DC-SIGNR plays a crucial role in MTCT of HIV-1 and that impaired placental DC-SIGNR expression increases risk of transmission.

## Introduction

Without specific interventions, the rate of HIV-1 mother-to-child transmission (MTCT) is approximately 15–45% [1]. UNAIDS estimates that last year alone, more than 400,000 children were infected worldwide, mostly through MTCT and 90% of them lived in sub-Saharan Africa. In the most heavily-affected countries, such as Zimbabwe, HIV-1 is responsible for one third of all deaths among children under the age of five. MTCT of HIV-1 can occur during pregnancy (in utero, IU), delivery (intrapartum, IP) or breastfeeding (postpartum, PP). High maternal viral load, low CD4 cells count, vaginal delivery, low gestational age have all been identified as independent factors associated with MTCT of HIV-1 [1]. Although antiretrovirals can reduce MTCT to 2%, limited access to timely diagnostics and drugs in many developing world countries limits the potential impact of this strategy. A better understanding of the mechanisms acting at the maternal-fetal interface is crucial for the design of alternative interventions to antiretroviral therapy for transmission prevention.

Dendritic cell-specific ICAM-grabbing non-integrin-related (DC-SIGNR, also known as CD209L or liver/lymph node-specific ICAM-grabbing non-integrin (L-SIGN)) can interact with a plethora of pathogens including HIV-1 and is expressed in placental capillary endothelial cells [2]. DC-SIGNR is organized in three distinct domains, an N-terminal cytoplasmic tail, a repeat region containing seven repeat of 23 amino acids and a C-terminal domain implicated in pathogen binding. Alternative splicing of DC-SIGNR gene leads to the production of a highly diversify isoforms repertoire which includes membrane-bound and soluble isoforms [3]. It has been proposed that interaction between DC-SIGNR and HIV-1 might enhance viral transfer to other susceptible cell types [2] but DC-SIGNR can also internalize and mediate proteasome-dependant degradation of viruses [4] that may differently affect the outcome of infection.

Given the presence of DC-SIGNR at the maternal-fetal interface and its interaction with HIV-1, we hypothesized that it could influence MTCT of HIV-1. To investigate the potential role of DC-SIGNR in MTCT of HIV-1, we carried out a genetic association study of DC-SIGNR in a well-characterized cohort of HIV-infected mothers and their infants recruited in Zimbabwe, and identified specific DC-SIGNR variants associated with increased risks of HIV transmission. We further characterized the functional impact of these genetic variants on DC-SIGNR expression and show that they affect both the level and type of DC-SIGNR transcripts produced in the placenta.

## Methods

### Subjects

Samples consisted of stored DNA extracts obtained from 197 mother-child pairs co-enrolled immediately postpartum in the ZVITAMBO Vitamin A supplementation trial (Harare, Zimbabwe) and followed at 6 weeks, and 3-monthly intervals up to 24 months. The ZVITAMBO project was a randomized placebo-controlled clinical trial that enrolled 14,110 mother-child pairs, between November 1997 and January 2000, with the main objective of investigating the impact of immediate postpartum vitamin A supplementation on MTCT of HIV-1. The samples used in the present study were from mother–child pairs randomly assigned to the placebo group of the ZVITAMBO project. Antiretroviral prophylaxis for HIV-1-positive antenatal women was not available in the Harare public-sector during ZVITAMBO patient recruitment. The samples were consecutively drawn from two groups: 97 HIV-1-positive mother/HIV-1-positive child pairs and 100 HIV-1-positive mother/HIV-negative child pairs. Mother's serological status was determined by ELISA and confirmed by Western Blot. Infants were considered to be infected if they were HIV-1 seropositive at 18 months or older and had two or more positive HIV-1-DNA polymerase chain reaction (PCR) results at earlier ages. 100 infants were considered to be uninfected as they were ELISA negative at 18 months or older and had two DNA PCR negative results from samples collected at a younger age. Of the 97 HIV-1-infected infants, 57 were infected IU, 11 were infected IP, and 17 were infected PP as determined by PCR analyses of blood samples collected at birth, 6 weeks, 3 and 6 months of age and according to the following definitions adapted from Bryson and colleagues [5]. Briefly, infants who were DNA PCR positive at birth were infected IU. Infants with negative PCR results from sample obtained at birth but who become positive by 6 weeks of age were infected IP. Infants with negative PCR results at birth and 6 weeks of age but who subsequently became DNA PCR positive were considered to be infected during the PP period. In the analysis comparing the 3 different modes of MTCT, 12 HIV-1-infected infants were excluded because the PCR results were not available at 6 weeks of age. Full methods for recruitment, baseline characteristics collection, laboratory procedures have been described elsewhere [6].

### DC-SIGNR haplotypes reconstruction, htSNPs selection and genotyping

**Figure 1:**
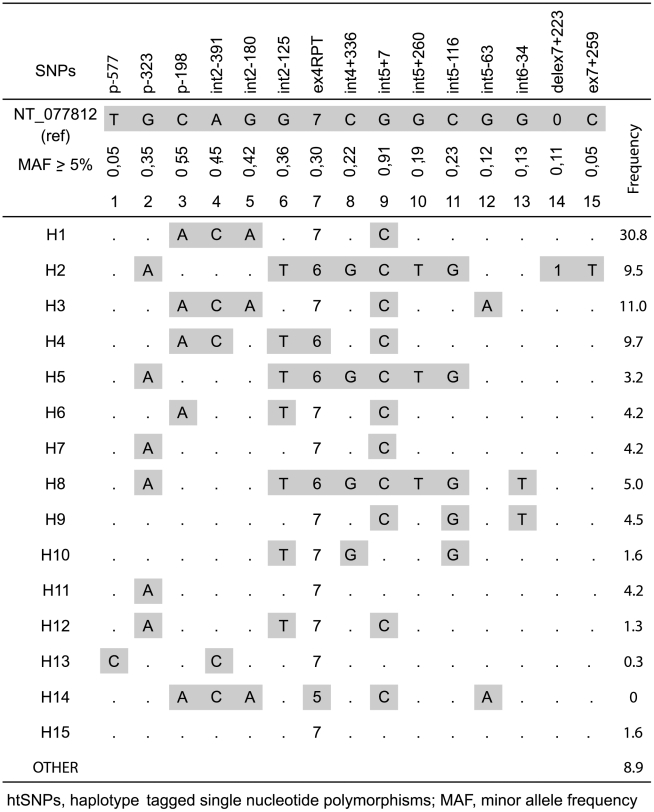
DC-SIGNR haplotypes reconstruction with minor allele frequency >5% (MAF) and selected htSNPs in Zimbabwean population.

The nucleotide sequence variation of the entire promoter, coding and part of 3′-UTR regions of DC-SIGNR gene in the study population was determined previously [7]. Haplotype reconstruction was performed using Bayesian statistical method implemented in PHASE [8], version 2.1.1, using single nucleotide polymorphism (SNP) with a minimum allele frequency (MAF) of 2%. We applied the algorithm five times, using different randomly generated seeds, and consistent results were obtained across runs (Figure 1). Fifteen haplotype-tagged SNPs (htSNPs) were identified by the HaploBlockFinder software [9] with a MAF ≥5%. These htSNPs were genotyped in the 197 infants by direct PCR sequencing analysis as we have described previously [7]. The DC-SIGNR exon 4 repeat region genotype was determined by PCR amplification followed by migration in 1.5% agarose gels [10]. DNA sequences in the promoter region were analysed with the TESS interface (http//:www.cbil.upenn.edu/tess) for putative transcription factors binding sites using the TRANSFAC database.

### Luciferase assays

Luciferase reporter assays using pGL2-Basic vector were performed in order to investigate the functional effect of mutations on DC-SIGNR promoter activity. Genomic DNA from subjects homozygous for the promoter variants and WT was amplified from nucleotide position −715 to −1 and cloned between the BglII and HindIII multiple cloning sites in the pGL2-Basic vector which harbours a reporter firefly luciferase gene downstream (Invitrogen Canada inc, Burlington, Canada). All recombinants clones were verified by DNA sequencing. The firefly luciferase test reporter vector was co-transfected at a ratio of 10∶1 with the constitutive expressor of Renilla luciferase, phRL-CMV (Promega, Madison, WI, USA). We cultured HeLa cells in 6 wells plates (2×10^5^ cells) and transfected them the following day using lipofectamine (Invitrogen) according to the manufacturer. Cells were lysed and luciferase assays were performed using 20 µg of protein extract according to the manufacturer (Promega) at 44 h post-transfection. Firefly luciferase activity was normalized to Renilla luciferase activity. 0 µg, 0,5 µg or 1 µg CMV-Tat vector was transfected with LTR-Luc as a positive control in these experiments. We carried out lucierase assays in triplicate in three independent experiments. Results are expressed as mean±standard error of the mean (S.E.M).

### DC-SIGNR isoforms repertoire

First-term placental tissues were obtained from abortions following voluntary interruption of pregnancy at CHUM Hôpital Saint-Luc (Montreal, Canada). Tissues from 3 H1 (associated with MTCT of HIV-1) and 3 H15 (wild-type) homozygous haplotypes were used to analyse possible differences in isoform expression. Total placental RNAs were extracted by MasterPure DNA and RNA Extraction Kit (Epicentre Biotechnologies, Madison, WI, USA) according to the manufacturer. Fragments corresponding to the DC-SIGNR coding region were reversed transcribed (RT) and then amplified by nested PCR with the following primers; RT primers RR, first PCR RF and RR and second PCR RcF and RcR according to Liu and colleagues [11]. 1 µg of total RNA was reverse transcribed with Expand RT (Roche Applied Science, Indianapolis, IN, USA) according to the manufacturer and were PCR-amplified with DNA Platinum Taq Polymerase (Invitrogen). Major PCR products from the second PCR reaction were gel extracted with the Qiagen Gel Extraction Kit (Qiagen Canada inc, Mississauga, ON, Canada) and cloned using the TOPO TA Cloning Kit for sequencing (Invitrogen). For each placenta, 15 different clones were randomly selected and amplified with M13 primers and sequenced with ABI PRISM 3100 capillary automated sequencer (Applied Biosystems, Foster City, CA, USA). Sequences were analysed and aligned with GeneBank reference sequence NM_014257 using Lasergene software (DNA Stars, Madison, WI, USA).

### Quantitative expression of DC-SIGNR isoforms

1,5 µg of placental RNA was reverse transcribed using 2.5 mM of Oligo dT_20_ and Expand RT in 20 µl volume according to the manufacturer (Roche Applied Science). 15 ng of total cDNA in a final volume of 20 µl was used to perform quantitative real-time PCR using Universal Express SYBR GreenER qPCR Supermix (Invitrogen) on a Rotor Gene Realtime Rotary Analyser (Corbett Life Science, Sydney, Australia). Samples from 2 subjects in each group were used because RNA quality of others was not suitable for a qRT-PCR analysis. Amplification of all DC-SIGNR isoforms was performed using an exon 5 specific primer pair (Table S1). Membrane-bound isoforms were amplified using primers specific for exon 3, corresponding to the common trans-membrane domain of DC-SIGNR. Primers were targeted to the exon-exon junction and RNA extracts were treated with DNase (Fermantas International inc, Burlington, ON, Canada) to avoid amplification of contaminant DNA. Standard curves (50–500 000 copies per reaction) were generated using serial dilution of a full-length DC-SIGNR or commercial GAPDH (Invitrogen) plasmid DNA. All qPCR reactions had efficiencies ranging from 99% to 100%, even in the presence of 20 ng of non-specific nucleic acids, and therefore could be compared. The copy number of unknown samples was estimated by placing the measured PCR cycle number (crossing threshold) on the standard curve. To correct for differences in both RNA quality and quantity between samples, the expression levels of transcripts were normalised to the reference GAPDH gene transcripts. GAPDH primer sequences were kindly provided by A. Mes-Masson at the CHUM. The results are presented as target gene copy number per 10^5^ copies of GAPDH. The ratio of membrane-bound isoforms was calculated as E3/E5. Soluble isoforms were calculated by subtracting membrane-bound from total isoforms. We carried out qPCR assays in triplicate in three independent experiments. Results are expressed as mean±S.E.M.

### Statistical analysis

Statistical analysis was performed using the GraphPad PRISM 5.0 for Windows (GraphPad Software inc, San Diego, CA, USA). Differences in baseline characteristics and genotypic frequencies of haplotypes or htSNPs were compared between groups using the χ^2^ analysis or Fisher's exact test. Logistic regression analysis was used to estimate odds ratios (OR) for each genotype and baseline risk factors. Multiple logistic regression was used to define independent predictors identified as significant in the crude analysis. ORs and 95% confidence interval were calculated with the exact method. Comparisons of continuous variables between groups were assessed with the unpaired two-tailed Student's *t* test when variables were normally distributed and with the Mann-Whitney U test when otherwise. Differences were considered significant at P<0.05.

### Ethics statement

Written informed consent was obtained from all mothers who participated in the study and the ZVITAMBO trial and the investigation reported in this paper were approved by The Medical Research Council of Zimbabwe, The Medicines Control Authority of Zimbabwe, The Johns Hopkins Bloomberg School of Public Health Committee on Human Research, and the CHUM and Montreal General Hospital Ethics Committees. First-term placental tissues were obtained from abortions following voluntary interruption of pregnancy at CHUM Hôpital Saint-Luc (Montreal, Canada) with written informed consent in accordance with the CHUM Research Ethics Committee.

## Results

We carried out an association study of DC-SIGNR polymorphism in 197 infants born to untreated HIV-1-infected mothers recruited in Harare, Zimbabwe. Among them, 97 infants were HIV-1-infected and 100 infants remained uninfected. Of the 97 HIV-1-infected infants, 57 were infected IU, 11 were infected IP, and 17 were infected PP. Timing of infection was not determined for 12 HIV-1-infected infants. Baseline characteristics of mothers and infants are presented in Table 1. Maternal age and CD4 cell count, child sex, mode of delivery, duration of membrane rupture and gestational age were similar among all groups. However, maternal viral load >29 000 copies/ml was associated with increased risk in both IU and PP with odds ratios (OR) of 3.64 (95% CI = 1.82–7.31, P = 0.0002) and 4.45 (95% CI = 1.50–13.2, P = 0.0045) for HIV-1 transmission, respectively.

**Table 1:** Baseline characteristics of mother and infants risk factors for intrauterine (IU), intrapartum (IP) and postpartum (PP) mother-to-child HIV-1 transmission.

Variables	HIV -	IU infection		IP infection		PP infection	
	% (N)	% (N)	OR (95% CI)	% (N)	OR (95% CI)	% (N)	OR (95% CI)
			P value		P value		P value
Maternal age, mean							
years	26	26		24		28	
(SD)	(25–27)	(24–27)	0.53^a^	(22–28)	0.40^a^	(25–31)	0.26^a^
Child's sex							
F	43 (42)	55 (31)	0.62 (0.32–1.20)	70 (7)	0.33 (0.08–1.34)	53 (8)	0.67 (0.22–1.99)
M	57 (55)	45 (25)	0.15^c^	30 (3)	0.18^b^	47 (7)	0.47^c^
Gestational age (weeks)							
≥37	95 (94)	98 (55)	0.34 (0.04–3.00)	80 (8)	4.70 (0.78–28.2)	94 (16)	1.18 (0.13–10.7)
<37	5 (5)	2 (1)	0.42^b^	20 (2)	0.12^b^	6 (1)	1.00^b^
Membrane rupture (hours)							
0 – 3	57 (56)	49 (26)	1.39 (0.71–2.71)	50 (5)	1.33 (0.36–4.91)	50 (8)	1.33 (0.46–3.84)
>3	43 (42)	51 (27)	0.34^c^	50 (5)	0.74^b^	50 (8)	0.60^b^
Mode of delivery							
Vaginal	87 (87)	93 (52)	0.52 (0.16–1.66)	90 (9)	0.74 (0.09–6.37)	82 (14)	1.43 (0.36–5.68)
Caesarean	13 (13)	7 (4)	0.30^b^	10 (1)	1.00^b^	18 (3)	0.70^b^
Maternal CD4 cell counts (cells/mm3)							
≥500	41 (35)	42 (20)	0.98 (0.48–2.01)	22 (2)	2.45 (0.48–12.5)	18 (3)	3.27 (0.87–12.2)
<500	59 (50)	58 (28)	0.96^c^	78 (7)	0.47^b^	82 (14)	0.098^b^
Maternal CD4/CD8 cells ratio							
≥0.5	51 (43)	52 (25)	0.94 (0.46–1.91)	56 (5)	0.82 (0.21–3.26)	35 (6)	1.88 (0.64–5.54)
<0.5	49 (42)	48 (23)	0.87^c^	44 (4)	1.00^b^	65 (11)	0.25^c^
Mothers' viral loads (copies/ml)							
≤29,000	71 (68)	40 (22)	3.64 (1.82–7.31)	64 (7)	1.39 (0.37–5.12)	35 (6)	4.45 (1.50–13.2)
>29,000	29 (28)	60 (33)	0.0002^c^	36 (4)	0.73^b^	65 (11)	0.005^c^

CI, Confidence interval; F, female; M, male; N, number; OR, odds ratio; SD, standard deviation.

a P value as determined by Mann-Whitney U test.

b P value as determined by the Fisher's exact test.

c P value as determined by the χ^2^ test.

Fifteen haplotype-tagged SNPs (htSNPs) corresponding to the 15 major DC-SIGNR haplotypes (Figure 1) described among Zimbabweans [7] were genotyped in our study samples (Tables S2 and S3). H1 (31%) and H3 (11%) were the most frequent haplotypes observed (Figure 1). Being homozygous for the H1 haplotype was associated with increased risk of both IU (OR: 4.42, P = 0.022) and PP (OR: 7.31, P = 0.016) HIV-1 transmission (Table 2). Infants harbouring two copy combinations of H1 and/or H3 haplotypes (H1-H1, H1-H3 or H3-H3) had increased risk of IU (OR: 3.42, P = 0.007) and IP (OR: 5.71, P = 0.025) but not PP (P = 0.098) HIV-1 infection compared to infant noncarriers (Table 2). The latter associations remained significant after adjustment was made for the maternal viral load for both IU (OR: 3.57, 95% CI = 1.30–9.82, P = 0.013) and IP (OR: 5.71, 95% CI = 1.40–23.3, P = 0.025) HIV-1 transmission. The H1 and H3 haplotypes share a cluster of mutations (p-198A, int2-391C, int2-180A, ex4RPT, int5+7C) (Figure 1). Of these, the p-198A and int2-180A variants were significantly associated with MTCT of HIV-1 (Table S2). In the unadjusted regression analysis, homozygous infants for the p-198A and int2-180A variants had increased risk of IU (OR: 2.07 P = 0.045, OR: 3.78, P = 0.003, respectively) and IP (OR: 2.47, P = 0.17, O.R: 5.71, P = 0.025, respectively) HIV-1 infection compared to heterozygote infants or noncarriers (Table 3). When adjustment was made for maternal factors, only the association with the int2-180A variant remained significant for IU (OR: 3.83, 95% CI = 1.42–10.4, P = 0.008) and IP (O.R: 5.71, 95% CI = 1.40–23.3, P = 0.025) HIV-1 transmission. Thus, infants homozygous for DC-SIGNR variant int2-180A contained in H1 and H3 haplotypes were 4-fold to 6-fold more likely to be infected by HIV-1 during pregnancy or at delivery, respectively.

**Table 2:** Associations between infant DC-SIGNR haplotypes and intrauterine (IU), intrapartum (IP) and postpartum (PP) mother-to-child HIV-1 transmission.

Child DC-SIGNR haplotype genotypes	HIV -	IU infection		IP infection		PP infection	
	% (N)	% (N)	OR (95% CI)	% (N)	OR (95% CI)	% (N)	OR (95% CI)
			P value		P value		P value
Haplotype H1							
H1-other/other-other	96 (95)	84 (43)	4.42 (1.26–15.5)	82 (9)	5.28 (0.85–32.9)	76 (13)	7.31 (1.63–32.8)
H1-H1	4 (4)	16 (8)	0.022^a^	18 (2)	0.11^a^	24 (4)	0.016^a^
Haplotype H3							
H3-other/other-other	99 (98)	98 (50)	1.96 (0.12–32.0)	100 (11)	NA	100 (17)	NA
H3-H3	1 (1)	2 (1)	1.00^a^	0	1.00^a^	0	1.00^a^
Haplotype H1-H3							
H1-other/H3-other/							
other-other	91 (90)	74.5 (38)	3.42 (1.35–8.68)	64 (7)	5.71 (1.40–23.3)	76 (13)	3.08 (0.83–11.5)
H1-H1/H1-H3/H3-H3	9 (9)	25.5 (13)	0.007^b^	36 (4)	0.025^a^	24 (4)	0.098^a^

CI, Confidence interval; N, number; NA, not applicable; OR, odds ratio.

a P value as determined by the Fisher's exact test.

b P value as determined by the χ^2^ test.

**Table 3:** Associations between infant DC-SIGNR promoter p-198 and intron 2 (int2)-180 variants and intrauterine (IU), intrapartum (IP) and postpartum (PP) mother-to-child HIV-1 transmission.

Child DC-SIGNR Mutations	HIV -	IU infection		IP infection		PP infection	
	% (N)	% (N)	OR (95% CI)	% (N)	OR (95% CI)	% (N)	OR (95% CI)
			P value		P value		P value
p-198							
CC/CA	75 (74)	59 (30)	2.07 (1.01–4.25)	54.5 (6)	2.47 (0.69–8.79)	59 (10)	2.07 (0.71–6.02)
AA	25 (25)	41 (21)	0.045^b^	45.5 (5)	0.17^a^	41 (7)	0.24^a^
int2-180							
GG/GA	91 (90)	72.5 (37)	3.78 (1.51–9.50)	64 (7)	5.71 (1.40–23.3)	76 (13)	3.08 (0.83–11.5)
AA	9 (9)	27.5 (14)	0.003^b^	36 (4)	0.025^a^	24 (4)	0.098^a^

CI, Confidence interval; N, number; OR, odds ratio.

a P value as determined by the Fisher's exact test.

b P value as determined by the χ^2^ test.

**Figure 2:**
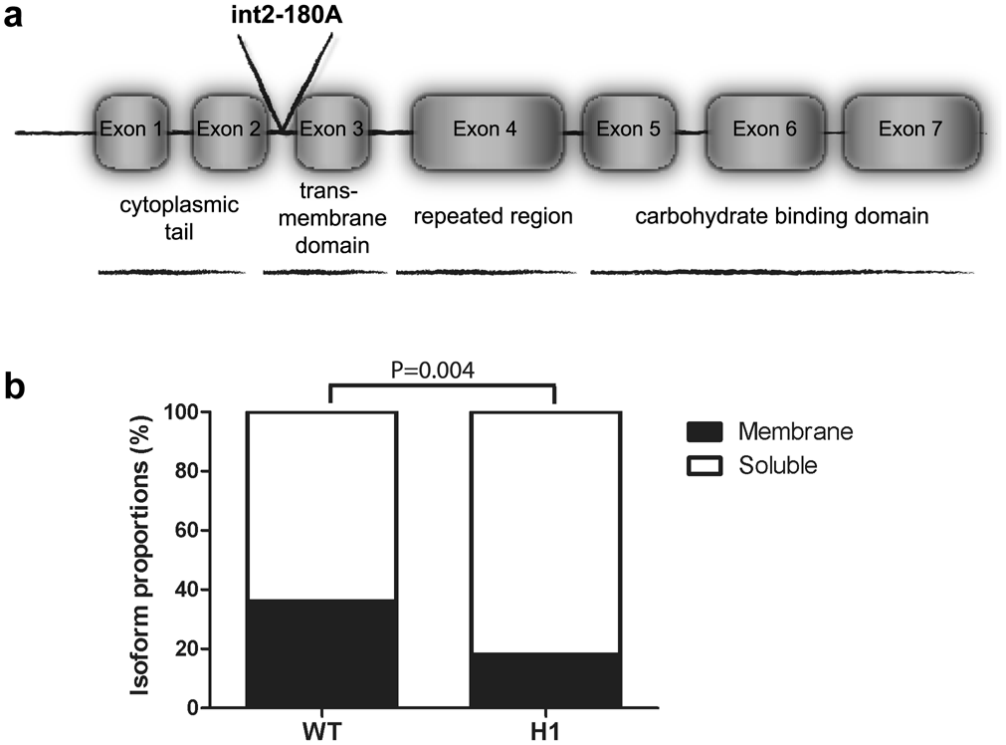
Effect of the int2-180A variant on placental DC-SIGNR isoform expression. (a) Schematic representation of the int2-180A variant location within the *DC-SIGNR* gene. (b) Proportion of DC-SIGNR membrane-bound and soluble isoforms in homozygous H1 (int2-180AA) and homozygous WT (int2-180GG) placental samples estimated by qRT-PCR assays in three independent experiments performed in triplicate. Data are presented as percentage of total transcripts. Differences between isoform proportions among the homozygous H1 and homozygous WT were calculated by the χ^2^ test.

Alternative splicing of the *DC-SIGNR* gene in the placenta produces both membrane-bound and soluble isoform repertoires [3]. The relative proportion of membrane bound and soluble DC-SIGNR could plausibly influence the susceptibility to HIV-1 infection [11]. We therefore hypothesized that the DC-SIGNR mutations associated with MTCT of HIV-1 would have an impact on both the level of DC-SIGNR expression and in the isoform repertoire produced. We investigated DC-SIGNR transcript expression in first-term placentas obtained after elective abortion. We cloned DC-SIGNR from placental tissues by RT-PCR from 3 homozygous H1 samples containing both the DC-SIGNR p-198AA and int2-180AA variants associated with HIV-1 transmission and 3 homozygous wild-type (WT) (p-198CC, int2-180GG) samples. Fifteen clones per sample were randomly selected for sequencing. As expected, we found an extensive repertoire of DC-SIGNR transcripts in all samples with 9 to 16 different isoforms per individual. A total of 65 distinct transcripts were identified (Figure S1), of which 3 were full-length transcripts. 64 of the sequenced clones contained a total of 69 amino acid substitutions with 3 new C termini and 2 premature stop codons. However, the diversity was mostly attributable to the entire deletion of exon 2 or exon 3 or to variations in the length of the neck region (exon 4) of DC-SIGNR. The deletion of exon 3 eliminates the trans-membrane domain of the protein and leads to the expression of soluble DC-SIGNR isoforms [3]. Interestingly, the abundance of membrane-bound isoforms in placental tissues of the H1 homozygotes appears to be lower than that observed in samples from WT individuals (Figure S1). The deletion of exon 3 was confirmed by sequencing and we hypothesize that the skipping of exon 3, could be due to the presence of the int2-180A mutation observed in infants with the H1 haplotype. In fact, this intron mutation is located 180 bp downstream from exon 3 and potentially modifies splicing events (Figure 2A). We confirmed that the variation in transcript proportions seen between the two groups was also reflected at the level of mRNA expression in the placenta. To quantify membrane-bound vs soluble isoforms in placental samples from homozygous H1 and WT infants, we amplified the exon 5 (E5) sequence present in all DC-SIGNR isoforms (total transcripts). We then amplified exon 3 (E3) which is deleted in the soluble forms and then calculated the E3∶E5 ratio. We found that placental tissues from homozygous H1 infants express a significantly lower proportion of membrane-bound DC-SIGNR (18%) compared to that in WT individuals (36%) (P = 0.004) (Figure 2B) suggesting that exon 3 skipping happens more frequently in presence of the DC-SIGNR int2-180A variant associated with MTCT of HIV-1.

**Figure 3:**
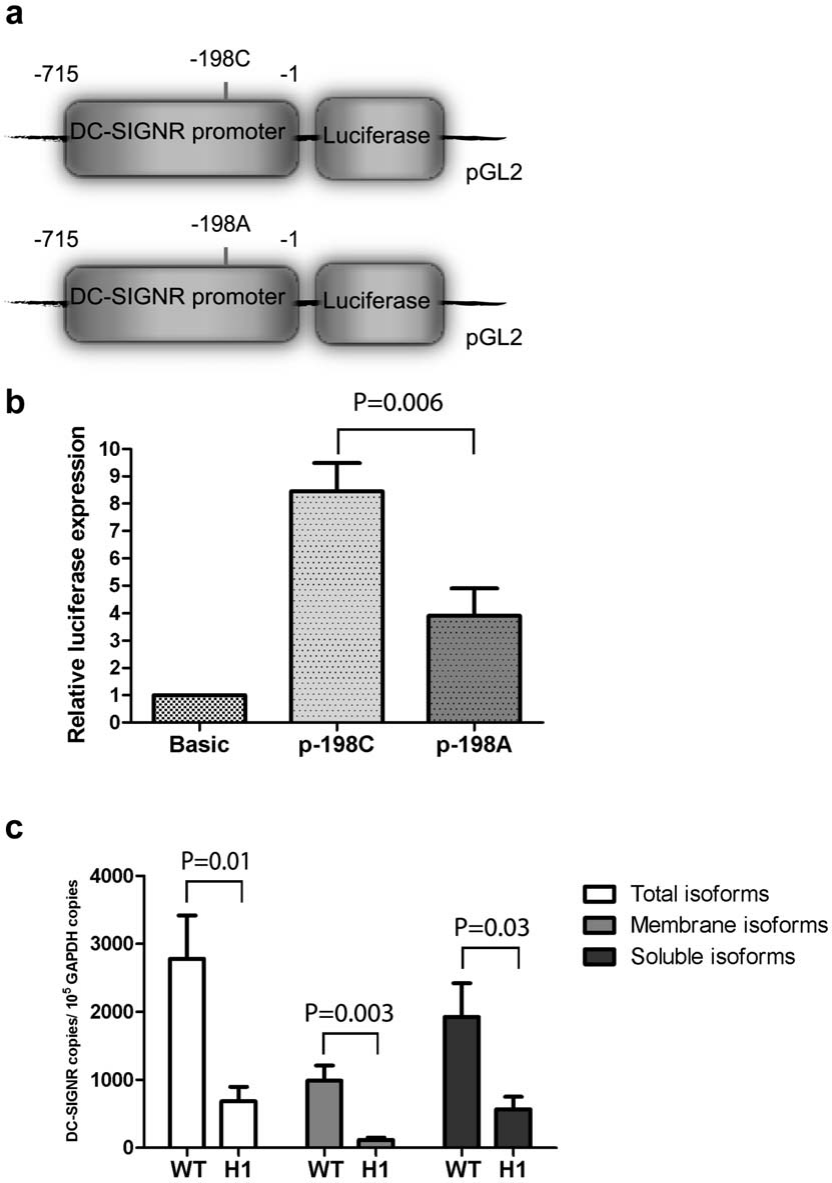
Transcriptional activity of DC-SIGNR promoters. (a, b) Effect of the p-198A variant on transcriptional activity in luciferase reporter assay *in vitro* in transfected HeLa cells. (a) Schematic representation of reporter gene constructs corresponding to the DC-SIGNR promoter region from –715 to –1, spanning with either C (WT) or A at position −198. (b) Relative luciferase expression from pGL2-Basic, the parental vector without the promoter. Expression of the DC-SIGNR promoter constructs were calculated relatively to this value. Data are presented in mean±S.E.M. values of three independent experiments performed in triplicate and difference in relative luciferase expression between the p-198 variants was examined with the Student's *t* test. (c) Total number of DC-SIGNR isoforms in homozygous H1 (p-198AA, int2-180AA) and homozygous WT (p-198CC, int2-180GG) placental samples measured by qRT-PCR assays in three independent experiments performed in triplicate. Data are shown in mean±S.E.M. copies number normalized by 10^5^ copies number of GAPDH. Student's *t* test was used to calculate differences in the number of isoforms between the H1 and WT groups.

The DC-SIGNR int2-180A variant is always transmitted with the promoter mutation p-198A (Figure 1). In the unadjusted regression analysis, the p-198A variant was significantly associated with IU but not with IP and PP HIV-1 transmission (Table 3). Computational transcription factor binding site analysis predicts that this mutation, located 198 bp upstream of the start codon would affect a C/EBPbeta transcription factor binding site (TESS web site). To test the effect of DC-SIGNR p-198A variant on transcription, we transiently transfected HeLa cells with a luciferase reporter gene under the control of *DC-SIGNR* promoter region –715 to –1 with either C (WT) or A at position -198 (Figure 3A). The luciferase activity of the p-198A variant construct was significantly lower than that of the WT p-198C promoter construct (p-198C/A ratio = 2, P = 0.006) (Figure 3B) suggesting that DC-SIGNR p-198A affects promoter activity. The other promoter mutants (p-577C and p-323A) observed in the Zimbabwean population did not affect DC-SIGNR transcription in this assay (Figure S2). To determine the net impact of the DC-SIGNR p-198A mutation on DC-SIGNR expression in the placenta, we quantitated the absolute number of total and membrane-bound DC-SIGNR transcripts in the H1 homozygote and wild-type placental samples as described earlier. The total number of DC-SIGNR transcripts was determined to be 685±213 (DC-SIGNR copies±S.E.M per 10^5^ GAPDH copies) in the placental samples from homozygous H1 infants and was 4-fold lower compared to that found in placentas from WT individuals (2781±638, P = 0.011) (Figure 3C). As suggested earlier, the int2-180A mutation might induce exon 3 skipping leading to a lower production of membrane-bound DC-SIGNR. Although, the decrease in the total number of DC-SIGNR transcripts in H1 homozygous placental samples containing both the p-198AA and int2-180AA variants affected the proportion of membrane-bound and soluble isoforms, the effect of these mutations was more pronounced on the membrane-bound isoforms with an 8-fold decrease (H1 = 117±36.2 vs WT = 990±220.6, P = 0.003) compared to a 3-fold decrease in total soluble isoforms (H1 = 568±181.9 vs WT = 1925±495.3, P = 0.03) (Figure 3C). Therefore, DC-SIGNR p-198A and int2-180A mutations associated with MTCT of HIV-1 significantly decreased the level of total placental DC-SIGNR transcripts, disproportionately affecting the membrane-bound isoform production.

## Discussion

Our genetic results, supported by expression assay in placenta, suggest the involvement of DC-SIGNR in MTCT of HIV-1. Homozygosity for the haplotype H1 was associated with IU transmission in the unadjusted regression analysis. However, the association disappeared after adjustment was made for the maternal factors presumably because of the small number of H1 homozygote infants analysed in each groups. H1 and H3 were the most frequent haplotypes observed in the study population and they share a cluster of mutations (Figure 1). Grouping haplotypes H1 and H3 increased the power of the study and permitted the identification of specific DC-SIGNR mutations associated with MTCT of HIV-1. Indeed, two mutations shared by haplotypes H1 and H3 were associated with vertical transmission of HIV-1. The int2-180A was associated with a 4-fold increased risk of IU and 6-fold increased risk of IP after adjustment for the maternal factors. Although the p-198A variant was associated with IU transmission, the association disappeared after adjustment was made for the maternal viral load. Nevertheless, we showed that this mutation reduces DC-SIGNR transcriptional activity *in vitro* and produces lower level of DC-SIGNR transcripts in placental tissues in combination with the int2-180A variant. Since int2-180A is always transmitted with p-198A on the MTCT associated combined haplotypes H1/H3, whereas p-198A is carried on other non-associated haplotypes (Figure 1), we can speculate that the p-198A mutation alone may have a minor effect *in vivo* whereas in combination with the int2-180A variant, they both act to reduce the level of placental DC-SIGNR expression resulting in an increased risk of MTCT of HIV-1.

The majority of IU transmission occurs during the last trimester of pregnancy (reviewed in [12]). Full-term placenta samples were not available for the current study and the expression assays were performed on first-term placental tissues. A previous study looking at DC-SIGNR placental isoforms repertoire in full-term placenta samples demonstrated similar diversity of DC-SIGNR transcripts as in the first-term placental tissues studied herein [3]. However, since levels of DC-SIGNR expression have never been compared between the different terms of pregnancy, it is not known whether DC-SIGNR expression varies during the course of pregnancy. Nevertheless, it is reasonable to assume that the inter-individual differences in both DC-SIGNR isoform repertoire and transcript levels observed between the H1 and WT homozygous infants would be reflected throughout the pregnancy.

To date, most studies have focused on the potential role of DC-SIGNR in *trans* infection of HIV-1 *in vitro* [2], [10]. However, the multiple mechanisms involved in *trans* infection and redundancy among C-type lectin functions make it difficult to determine the actual participation of DC-SIGNR in this mode of infection *in vivo* [13], [14]. The strong correlation we observed between MTCT of HIV-1 and DC-SIGNR genetic variants producing low levels of DC-SIGNR in the placenta suggested that mechanisms other than DC-SIGNR-mediated *trans* infection might operate during vertical transmission of HIV-1. For example, DC-SIGNR has also been shown to function as a HIV-1 antigen-capturing receptor [15]. Chan and colleagues recently demonstrated that DC-SIGNR transfected CHO cells diminish SARS-CoV titers by enhanced capture and degradation of the virus in a proteasome-dependent manner [4]. Since endothelial cells express MHC-I and II, degraded viral antigens could then be presented to immune cells to elicit an adaptive immune response [16], [17]. The HIV-1 co-receptor CCR5, but not CD4, is co-expressed with DC-SIGNR on placental and blood-brain barrier (BBB) endothelial cells [18], [19]. HIV-1 gp120 binding to CCR5 receptor on endothelial cells compromises BBB integrity and enhances monocytes adhesion and transmigration across the BBB [20], [21]. It is thus possible that reduced expression of DC-SIGNR, particularly the membrane-bound isoforms, on placental capillary endothelial cells might favour HIV-1 binding to CCR5 receptor, instead of DC-SIGNR receptor, facilitating the migration of maternal HIV-1-infected cells across the placental barrier resulting in IU transmission of HIV-1.

The int2-180A variant contained in the H1 and H3 haplotypes was associated with IP transmission suggesting that DC-SIGNR also affect transmission of HIV-1 during delivery. Little is known about the mechanisms underlying transmission of HIV-1 during delivery. Passage through the birth canal could potentially expose infants through a mucosal portal entry (presumably ophthalmic, skin, or gastrointestinal), whereas placental insult during delivery (physical or inflammatory) may enhance transplacental passage of maternal HIV-1-infected cells into foetal circulation [22], [23]. Such process called microtransfusion has been proposed in regards to the results obtain in a Malawian cohort. Kweik and colleagues found a significant association between levels of maternal DNA in umbilical cord blood and IP transmission of HIV-1 suggesting that passage of maternal infected cells through the placenta is likely to occur during delivery [22]. Thus, in a similar fashion as suggested earlier for IU transmission, the relatively lower level of DC-SIGNR in the placenta of homozygous infants harbouring the int2-180A variant could promote HIV-1 binding to CCR5 receptor on endothelial cells affecting the placental barrier integrity and facilitating the passage of maternal infected cells in foetal circulation during delivery.

Beside DC-SIGNR, other HIV-1 receptors are known to influence MTCT of HIV-1 (reviewed in [24]). Genetic variants in CCR5 have been shown to influence vertical transmission of HIV-1. CCR5 promoter variants resulting in higher expression of the receptor were associated with increased risk of MTCT of HIV-1 among sub-Saharan Africans [25], [26]. The 32-pb deletion polymorphism in CCR5 has be shown to protect from vertical transmission of HIV-1 [27], but this variant is virtually absent among African populations [28]. High copy numbers of CCL3L1, a potent HIV-1 suppressive ligand for CCR5, are associated with higher chemokine production and lower risk of MTCT of HIV-1 among South African infants [29], [30]. Mannose-binding lectin (MBL) is an innate immune receptor synthesised in the liver and secreted in the bloodstream in response to inflammation signal. MBL promotes pathogen elimination by opsonization and phagocytosis, and reduced expression of MBL resulting from polymorphism in coding and non-coding regions has been associated with an increased risk of MTCT of HIV-1 [31], [32].

In this study, we demonstrate for the first time, the potential functional impact of DC-SIGNR mutations on its expression in the placenta and in vertical transmission of HIV-1. We believe that the presence of DC-SIGNR at the placental endothelial cell surface may protect infants from HIV-1 infection by capturing virus and promoting its degradation/presentation. However, in placenta containing low levels of DC-SIGNR, HIV-1 would preferentially binds CCR5 on endothelial cells resulting in a loss of placental barrier integrity and enhanced passage of maternal HIV-1-infected cells in foetal circulation leading to MTCT of HIV-1. This mechanism may also apply to other vertically-transmitted pathogens known to interact with DC-SIGNR such as HIV-2, hepatitis C and dengue viruses and warrant further investigation.

## Supporting Information

Table S1 Primer pairs and qRT-PCR conditions for DC-SIGNR expression assays. a Accession number NM_014257, b Accession number NM_002046
(0.03 MB DOC)

Table S2 Associations between child DC-SIGNR htSNPs and mother-to-child HIV-1 transmission. CI, Confidence interval; htSNPs, haplotypes-tagged single nucleotide polymorphisms; N, number; OR, odds ratio, NA; not applicable, Del; deletion. a P-value as determined by the Chi-square test.
(0.09 MB DOC)

Table S3 Associations between child DC-SIGNR exon 4 repeated region genotypes and mother-to-child HIV-1 transmission.CI, Confidence interval; N, number; NA; not applicable; OR, odds ratio a P-value as determined by the Chi-square test. b Comparison between genotype and all others.
(0.05 MB DOC)

Figure S1 DC-SIGNR transcripts repertoire in placenta. Major RT-PCR products from RNA extract from 3 homozygous H1 and 3 homozygous WT placenta samples were purified, cloned and sequenced. Sequenced were analysed according to NCBI reference sequence NM_014257. CT; cytoplasmic tail, TM; trans-membrane domain; WT; wild-type
(0.11 MB DOC)

Figure S2 Effect of DC-SIGNR promoter variant on transcriptional activity in luciferase reporter assay in vitro in transfected HeLa cells. Relative luciferase expression from pGL2-Basic, parental vector without promoter. Expression DC-SIGNR promoter constructs, spanning p-577C variant or p-323A variant were calculated relatively to this value. Data are presented in mean values±S.E.M of three independent experiments performed in triplicate. One-way ANOVA test followed by the Dunnett test for multiple comparison was used to compare the relative luciferase expression of the p-557C and p-323A variant reporters against the wild-type (WT) construct (not significant). 0 µg, 0,5 µg or 1 µg CMV-Tat vector was transfected with LTR-Luc as a positive control in these experiments.
(0.27 MB DOC)
