# Supplementary material for: Functional Genetic Variants in DC-SIGNR Are Associated with Mother-to-Child Transmission of HIV-1
Source: PLoS One. 2009 Oct 7;4(10):e7211. doi: 10.1371/journal.pone.0007211 (PMC2752805; doi:10.1371/journal.pone.0007211)
Supplement: Table S1 — Primer pairs and qRT-PCR conditions for DC-SIGNR expression assays. a Accession number NM_014257, b Accession number NM_002046 (0.03 MB DOC) [file pone.0007211.s001.doc]

| Gene |  | Primer pair | Annealing T° | Product size |
| --- | --- | --- | --- | --- |
| DC-SIGNR-Exon 5^a^ | Total isoforms | SRE4/5F : 5’-CTGCATTTGGAACGCCTGTGC-3’ SR5R : 5’-GCTCCTCAGCAGTTTTGATTACG-3’ | 63°C | 151 bp |
| DC-SIGNR-Exon 3^a^ | Membrane-bound isoforms | SR3F : 5’-CCCTGGTGCAACTCCTCTC-3’ SRE3/4R : 5’-GACCTTGGACACTTGGACAAGGATG-3’ | 63°C | 67 bp |
| GAPDH^b^ | Reference | GAPDHF : 5'-CGGGAAGCTCACTGGCATGGC-3' GAPDHR : 5'-GGTGGAGGAGTGGGTGTCGCTGTT-3' | 60°C | 208 bp |
