# Supplementary material for: Functional Genetic Variants in DC-SIGNR Are Associated with Mother-to-Child Transmission of HIV-1
Source: PLoS One. 2009 Oct 7;4(10):e7211. doi: 10.1371/journal.pone.0007211 (PMC2752805; doi:10.1371/journal.pone.0007211)
Supplement: Table S2 — Associations between child DC-SIGNR htSNPs and mother-to-child HIV-1 transmission. CI, Confidence interval; htSNPs, haplotypes-tagged single nucleotide polymorphisms; N, number; OR, odds ratio, NA; not applicable, Del; deletion. a P-value as determined by the Chi-square test. (0.09 MB DOC) [file pone.0007211.s002.doc]

| Child DC-SIGNR htSNPs genotypes | HIV - | HIV+ |  |
| --- | --- | --- | --- |
|  | % (N) | % (N) | OR (95% CI) |
|  |  |  | P value^a^ |
|  |  |  |  |
| p-577 |  |  |  |
| TT | 94.9 (94) | 100 (91) | NA |
| CT | 0.05 (5) | 0 |  |
|  |  |  |  |
| p-323 |  |  |  |
| GG/AG | 85.9 (85) | 89.0 (81) | 0.75 (0.32-1.78) |
| AA | 14.1 (14) | 11.0 (10) | 0.514 |
|  |  |  |  |
| p-198 |  |  |  |
| CC/CA | 74.7 (74) | 60.4 (55) | 1.94 (1.04-3.60) |
| AA | 25.2 (25) | 39.6 (36) | 0.035 |
|  |  |  |  |
| int2-391 |  |  |  |
| AA/CA | 75.8 (75) | 65.9 (60) | 1.62 (0.86-3.04) |
| CC | 24.2 (24) | 31.3 (31) | 0.136 |
|  |  |  |  |
| Int2-180 |  |  |  |
| GG /GA | 90.9 (90) | 73.6 (67) | 3.58 (1.56-8.21) |
| AA | 9.1 (9) | 26.4 (24) | 0.002 |
|  |  |  |  |
| int2-125 |  |  |  |
| GG /GT | 85.8 (85) | 87.9 (80) | 0.84 (0.36-1.95) |
| TT | 15.4 (14) | 12.1 (11) | 0.676 |
|  |  |  |  |
| int4+336 |  |  |  |
| CC/CG | 96.0 (95) | 96.7 (88) | 0.81 (0.18-3.72) |
| GG | 4.0 (4) | 3.3 (3) | 0.786 |
|  |  |  |  |
| int5+7 |  |  |  |
| GG/CG | 24.2 (24) | 19.8 (18) | 1.3 (0.65-2.59) |
| CC | 75.8 (75) | 80.2 (73) | 0.459 |
|  |  |  |  |
| int5+260 |  |  |  |
| GG/GT | 96 (95) | 98.9 (90) | 0.26 (0.03-2.41) |
| TT | 4 (4) | 1.1 (1) | 0.206 |
|  |  |  |  |
| int5-116 |  |  |  |
| CC/CG | 90.9 (90) | 93.4 (85) | 0.71 (0.24-2.07) |
| GG | 9.1 (9) | 6.6 (6) | 0.524 |
|  |  |  |  |
| int5-63 |  |  |  |
| GG/GA | 98.0 (97) | 98.9 (90) | 0.54 (0.05-6.05) |
| AA | 2.0 (2) | 1.1 (1) | 0.611 |
|  |  |  |  |
| int6-34 |  |  |  |
| GG/GT | 97 (96) | 98.9 (89) | 0.36 (0.04-3.52) |
| TT | 3 (3) | 1.1 (1) | 0.360 |
|  |  |  |  |
| ex7del+223 |  |  |  |
| TCT/ Del/TCT | 100 (99) | 100 (91) | NA |
| Del/Del | 0 | 0 |  |
|  |  |  |  |
| ex7+259 |  |  |  |
| CC/CT (ref) | 100 (99) | 100 (91) | NA |
| TT | 0 | 0 |  |
|  |  |  |  |
