# Supplementary material for: Functional Genetic Variants in DC-SIGNR Are Associated with Mother-to-Child Transmission of HIV-1
Source: PLoS One. 2009 Oct 7;4(10):e7211. doi: 10.1371/journal.pone.0007211 (PMC2752805; doi:10.1371/journal.pone.0007211)
Supplement: Table S3 — Associations between child DC-SIGNR exon 4 repeated region genotypes and mother-to-child HIV-1 transmission.CI, Confidence interval; N, number; NA; not applicable; OR, odds ratio a P-value as determined by the Chi-square test. b Comparison between genotype and all others. (0.05 MB DOC) [file pone.0007211.s003.doc]

| Child DC-SIGNR ex4RPT genotypes | HIV - | HIV+ |  |
| --- | --- | --- | --- |
|  | % (N) | % (N) | OR (95% CI) |
|  |  |  | P value^a^ |
|  |  |  |  |
| 8/7^b^ | 3.0 (3) | 3.3 (3) | 1.09 (0.21-5.55) |
|  |  |  | 1.00 |
|  |  |  |  |
| 8/6^b^ | 1.0 (1) | 1.1 (1) | 1.09 (0.07-17.68) |
|  |  |  | 1.00 |
|  |  |  |  |
| 7/7^b^ | 44.4 (44) | 50.5 (46) | 0.78 (0.44-1.39) |
|  |  |  | 0.400 |
|  |  |  |  |
| 7/6^b^ | 36.4 (36) | 34.1 (31) | 0.92 (0.50-1.69) |
|  |  |  | 0.192 |
|  |  |  |  |
| 7/5^b^ | 0 | 1.1 (1) | NA |
|  |  |  |  |
| 6/6^b^ | 15.2 (15) | 6.6 (6) | 0.39 (0.14-1.07) |
|  |  |  | 0.060 |
|  |  |  |  |
| 6/5^b^ | 0 | 3.3 (3) | NA |
|  |  |  |  |
| homozygote | 59.6 (59) | 57.1 (52) | 1.11 (0.62-1.97) |
| heterozygote | 40.4 (40) | 42.9 (39) | 0.732 |
|  |  |  |  |
