# Supplementary figures and images for: Functional Genetic Variants in DC-SIGNR Are Associated with Mother-to-Child Transmission of HIV-1
Source: PLoS One. 2009 Oct 7;4(10):e7211. doi: 10.1371/journal.pone.0007211 (PMC2752805; doi:10.1371/journal.pone.0007211)

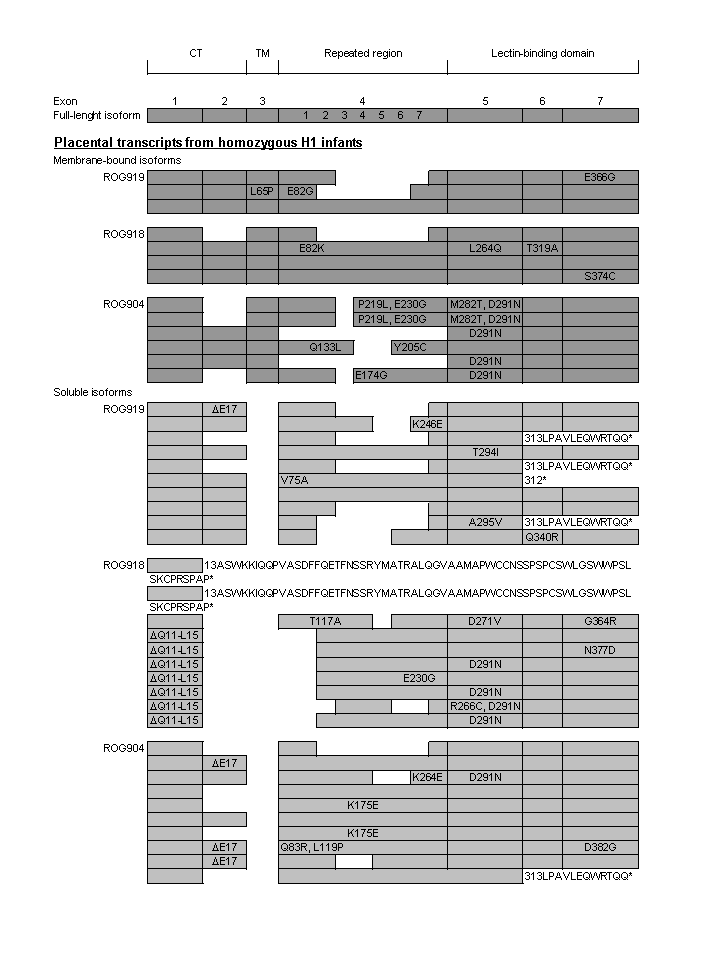


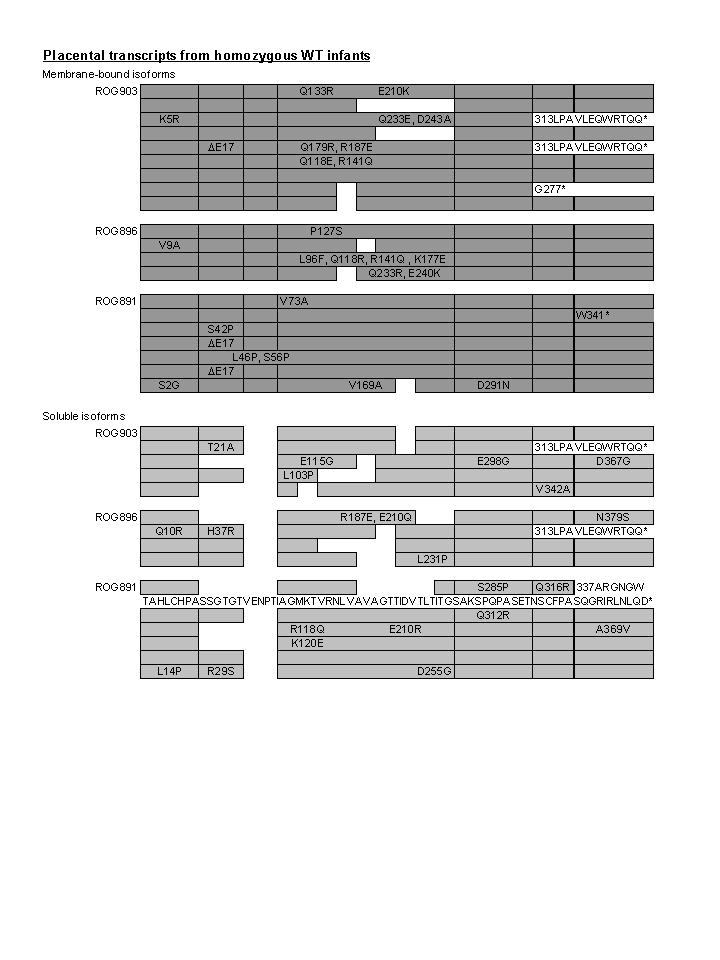

Supplement: Figure S1 — DC-SIGNR transcripts repertoire in placenta. Major RT-PCR products from RNA extract from 3 homozygous H1 and 3 homozygous WT placenta samples were purified, cloned and sequenced. Sequenced were analysed according to NCBI reference sequence NM_014257. CT; cytoplasmic tail, TM; trans-membrane domain; WT; wild-type (0.11 MB DOC) [file pone.0007211.s004.doc]

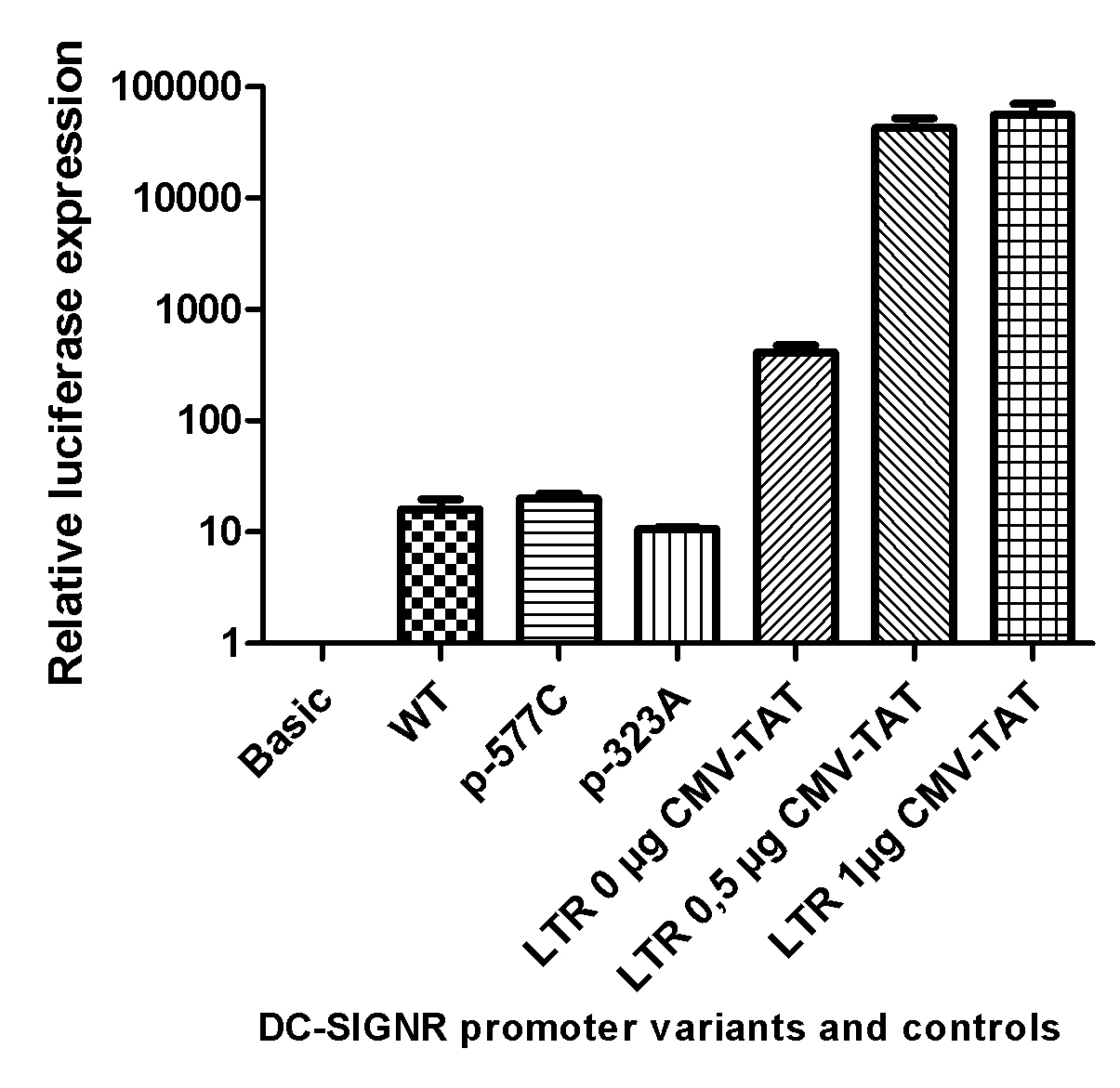

Supplement: Figure S2 — Effect of DC-SIGNR promoter variant on transcriptional activity in luciferase reporter assay in vitro in transfected HeLa cells. Relative luciferase expression from pGL2-Basic, parental vector without promoter. Expression DC-SIGNR promoter constructs, spanning p-577C variant or p-323A variant were calculated relatively to this value. Data are presented in mean values±S.E.M of three independent experiments performed in triplicate. One-way ANOVA test followed by the Dunnett test for multiple comparison was used to compare the relative luciferase expression of the p-557C and p-323A variant reporters against the wild-type (WT) construct (not significant). 0 µg, 0,5 µg or 1 µg CMV-Tat vector was transfected with LTR-Luc as a positive control in these experiments. (0.27 MB DOC) [file pone.0007211.s005.doc]
